# Gossypol Promotes Wnt/*β*-Catenin Signaling through WIF1 in Ovariectomy-Induced Osteoporosis

**DOI:** 10.1155/2019/8745487

**Published:** 2019-04-21

**Authors:** Jinqian Liang, Chong Chen, Hongzhe Liu, Xiangyang Liu, Hong Zhao, Jianhua Hu

**Affiliations:** ^1^Department of Orthopaedic Surgery, Peking Union Medical College Hospital, Beijing 100730, China; ^2^Department of Orthopedics, People's Hospital of Hunan Province, Hunan 410005, China

## Abstract

Osteoporosis is one of the most frequent diseases related with age. Previously, we have reported a novel potential drug, gossypol, for the treatment of osteoporosis through its regulation of Wnt/*β*-catenin signaling. This study aims to identify the detailed mechanism of gossypol in human osteoporosis. Mice injected with gossypol were subjected for RNA-seq analysis and the transcription level of WIF1 was shown to be decreased dramatically in gossypol-treated mice, which was further confirmed by qRT-PCR and western blot analysis. Luciferase reporter assay showed gossypol inhibited the activity of WIF1 and the methylation of WIF1 was significantly upregulated, evidenced by ChIP assay. Cell viability assays demonstrated that gossypol promoted cell proliferation while cotreatment with WIF1 expressing plasmid reversed the effect in a dose- and time-dependent manner. Similarly, cell apoptotic assays and TUNEL assays showed gossypol suppressed cell apoptosis, which was revised by WIF1 overexpression. The mouse model suggested gossypol injection ameliorated osteoporosis, while coinjection of AAV5-WIF1 eliminated the protection effects of gossypol, as evidenced by H&E staining, serum osteocalcin level, serum OPG level, serum RANKL level, bone density, ultimate strength, and postyield displacement. This study is a supplement to the former publication, which reinforced the protection effect of gossypol in human osteoporosis.

## 1. Introduction

Osteoporosis is a bone disease that occurs when too much bone is lost or too little bone is made, or both. Osteoporosis bones have lost density or mass and contain abnormal bone structures. The healthy bone looks like a honeycomb when viewed under a microscope; the osteoporosis bones have much larger holes and spaces in the honeycomb [1]. Approximately, 54 million Americans have osteoporosis and almost 1.5 million fractures per year are caused by osteoporosis, with the vast majority occurring in postmenopausal females [2]. It is estimated that more than a half of worldwide osteoporosis hip fractures will occur in Asia by 2050 [3, 4] and the white women will have a 15 to 20% lifetime risk of hip fracture when they are older than 50 [5]. Hip fractures can result in poor quality of life and increasing risk of death. The overriding goal is to prevent future fractures for postmenopausal osteoporosis [6]. Therefore, to identify a novel small molecule and elucidate the detailed mechanisms for osteoporosis are a clinical priority [7].

Ovarian hormone deficiency following ovariectomy is a major risk for osteoporosis females, which causes an acute decrease of circulating ovarian hormones, such as E2, resulting in “surgical menopause,” distinct from the natural menopause, during which the loss of hormones is gradual [8]. Nowadays, several of pharmacological treatments have been brought into clinic, including hormone therapy, bisphosphonate, and selective estrogen receptor; however, the side effects are also serious. Thus, natural compound with antiosteoporotic activity and fewer side effects is worthy of exploring.

In our previous study, we identified a new chemical, gossypol, to prevent osteoporosis by regulating cell proliferation and apoptosis [9]. Furthermore, Wnt signaling was demonstrated to be promoted by gossypol treatment in vivo and in vitro. However, the detailed mechanism remained uncovered. Herein, we dig further to proclaim the underlying mechanism.

## 2. Materials and Methods

### 2.1. Animals

Some of the materials and methods were following the previous publication [9]. This study was approved by the Ethics Committee of Department of Orthopedic, People's Hospital of Hunan Province. An amount of 40 C57BL/6 mice (age: 8 weeks, body weight: 20g, female) were from Model Animal Research Center of Nanjing University. After adaption for 1 week, all of the mice underwent oophorectomy and were randomly divided into four groups: control group (*i.p*. injection of DMSO and* i.v. *injection of AAV5-GFP), gossypol group (*i.p.* injection of gossypol and* i.v. *injection of AAV5-GFP), AAV5-WIF1 group (*i.p.* injection of DMSO and* i.v. *injection of AAV5-WIF1), and gossypol+AAV5-WIF1 (*i.p.* injection of gossypol and* i.v. *injection of AAV5-WIF1). Gossypol was commercially purchased from APExBIO Co. (Catalog number: N2135, NY, USA) and dissolved in DMSO with a concentration of 15mg/ml. AAV5-WIF1 or its negative control AAV5-GFP was purchased from HanHeng Co. (Wuhan, China) and its titer was 1×10^12^. The injection of AAV5 was performed once and then the injection of gossypol was continued every day for four weeks. Afterwards, mice were sacrificed after anesthesia with 7% pentobarbital sodium and the thighbone of each mouse was harvested.

### 2.2. Cell Culture and Transfection

MC3T3-E1 cell line was purchased from American Type Culture Collection (ATCC, CRL-2593, Massachusetts, USA) and cultured in DMEM (Gibco, NY, USA) supplemented with 10% fetal bovine serum (FBS, Gibco). Cells were treated with gossypol with a concentration of 20nm for 24h, unless otherwise stated. Cell transfection was performed with Lipofectamine 2000 as per the manufactures' instructions. WIF expressing plasmid was purchased from Addgene Co. (#99533, NY, USA).

### 2.3. RNA-Seq Analysis

Thighbones from mice injected with gossypol and control vehicle (n=5) were subjected for RNA-seq analysis by BGI Co. (Shanghai, China). The genes upregulated and downregulated significantly were analyzed and those involved in Wnt signaling were presented.

### 2.4. qRT-PCR Analysis

Total RNAs from cells and tissues were extracted with TRIzol reagent (Thermo Scientific, NY, USA). RNAs were quantified by NanoDrop 2000 and a total of 1mg RNA were reversely transcribed with Prime Script TM Master Mix (TakaRa, Dalian, China). qRT-PCR was performed with SYBR Premix EX Taq TM II (Takara) on ABI7500 (Thermo Fisher Scientific, CA, USA). GAPDH was included as an inner control. The qPCR protocol was shown as below: initial denaturation at 95°C for 5 min, followed by 45 repeats of a three-step cycling program consisting of 10 sec at 95°C (denaturation), 10s at 60°C (primer annealing), and 10 sec at 72°C (elongation), and a final extension step for 10 min at 72°C. The primers used were listed here: WIF1: Forward: 5'-TCTGGAGCATCCTACCTTGC-3' and Reverse: 5'-ATGAGCACTCTAGCCTGATGG-3'; SFRP: Forward: 5'- CGTGGGCTCTTCCTCTTCG-3' and Reverse: 5'- ATGTTCTGGTACTCGATGCCG-3'; DKK1: Forward: 5'- CTCATCAATTCCAACGCGATCA and Reverse: 5'- GCCCTCATAGAGAACTCCCG-3'; GAPDH: Forward: 5'- GTGGACATCCGCAAAGAC-3' and Reverse: 5'- AAAGGGTGTAACGCAACTA-3'.

### 2.5. Western Blot Analysis

Proteins were extracted from mouse model with lysis buffer (NP-40, Beyotime, Nantong, China) and a total of 20*μ*g proteins were loaded up to a 10% SDS-PAGE gel. After being transferred to a nitrocellulose membrane (NC membrane, Millipore, USA), the proteins were blocked with 5% skim milk at room temperature for 1 hour and incubated with primary antibodies at 4°C overnight. The primary antibodies against WIF1 (ab-155101, 1:1000), SFRP (ab-137560, 1:1000), and DKK1 (ab-61275, 1:1000) were purchased from Abcam (Cambridge, USA). The primary antibodies GAPDH (sc-47724) and secondary antibodies (1:5000) were commercially purchased from Santa Cruz Biotechnology (Santa Cruz, CA, USA).

### 2.6. Luciferase Reporter Assay

The promoter sequence of WIF-1 was cloned into pGL3 vector with XhoI and HindIII restrictive enzyme sites. After DNA sequencing, the right plasmids were transfected into 293T cells together with Renilla control plasmids. Two days after transfection, Dual Luciferase Reporter Assay System (Promega, E1910, USA) was used. Firefly luciferase activity was normalized to that of Renilla for each experimental group.

### 2.7. Chromatin Immunoprecipitation (ChIP)

A ChIP assay was conducted using ChIP™ (17-371, Millipore) as per the manufacturer's instructions. Briefly, the DNA extracts with or without gossypol treatment were fragmented with sonication and pulled down with H3K27 antibody and subjected to the amplification by PCR. The potential binding site on the promoter of WIF-1 was detected by both PCR and qPCR reactions.

### 2.8. Cell Viability Assays

Cell viability was assessed with the methylthiazol tetrazolium (MTT) assay (Beyotime, Nantong, China). Briefly, cells were treated with gossypol (20 nM) and for 24h in the presence or absence of WIF overexpression. 48 hours later, cells were added with 10*μ*l of MTT solution (5 mg/mL) per well and incubated for 2h at 37°C incubator; the absorbance of each well was recorded at 450 nm in triplicate.

### 2.9. Apoptotic Analysis

The Annexin V/PI assay was conducted according to the manufacturer's instructions (Beyotime). Briefly, cells were transfected with WIF expressing plasmid and then treated with gossypol for 24 hours at 37°C. Afterwards, cells were washed with prechilled PBS, trypsinized for 1 min, and resuspended in 100*μ*l of binding buffer supplemented with 2.5*μ*l FITC conjugated Annexin-v and 1*μ*l PI (100 *μ*g/ml). Then, cells were shaken at room temperature for 15 min in darkness. A total of 10, 000 cells were collected and assessed by flow cytometry (BD Biosciences).

### 2.10. Terminal Deoxynucleotidyl Transferase dUTP Nick End Labeling (TUNEL) Assays

The TUNEL assays were performed according to the protocols (Vazyme, Nanjing, China). A coverslip was placed into 12-well plate and cells were seeded into each well. Cells were transfected with WIF expressing plasmid and then stimulated with gossypol for 24 hours. After incubation with lysis buffer for 10 min on ice, the cell pellet was collected with low-speed centrifugation (1000g, 5min, 4°C). Slides with adherent cells were covered with 50ul terminal deoxynucleotidyl transferase reaction mixture for 60 min at 37°C to avoid light. After being stopped with SSC, nuclei were stained and visualized with DAPI staining for 15 min at room temperature. Five random fields were photographed with the Nikon light microscope (400×).

### 2.11. Hematoxylin and Eosin (H&E) Staining

The thighbone from mouse model was fixed in 10% paraformaldehyde and decalcified. After being dehydrated, the tissues were embedded with paraffin and cut into 5*µ*m slices for hematoxylin and eosin (H&E) staining. Five random fields were selected and photographed under a Nikon (Japan, 200×) light microscope to calculate bone formation and osteoporosis.

### 2.12. ELISA Assays

Blood from each mouse was collected for biochemical analyses. The serum osteocalcin levels were assessed with a Life Technology enzyme-linked immunosorbent assay (ELISA, CA, USA) kit according to the manufactures' instructions. Serum RANKL and osteoprotegerin (OPG) levels were determined with ELISA kits from R&D system (Minneapolis, MN, USA) as per the protocols.

### 2.13. Statistical Analysis

GraphPad Prism 5.0 (GraphPad Software, La Jolla, CA, USA) software was used for statistical analysis. Data were shown as mean ± standard deviation (SD). Differences were considered significant when a two-sided* p *value was less than 0.05.

## 3. Results

### 3.1. Wnt Signaling Was Upregulated by Gossypol Treatment* In Vivo*

**Figure 1:**
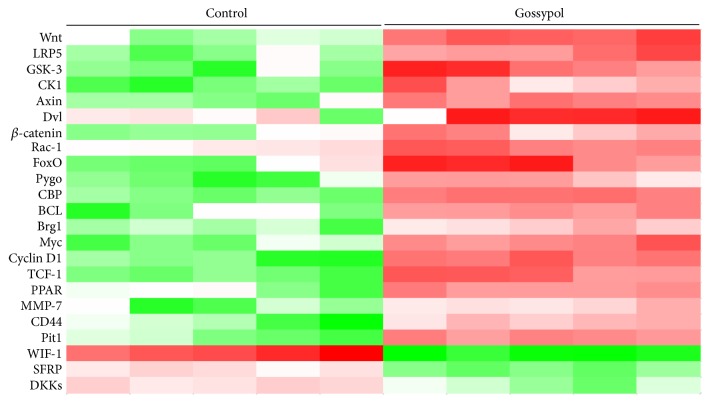
*Wnt signaling was upregulated by gossypol treatment in vivo*. RNA-seq analysis was performed in ovariectomy-induced osteoporosis mice injected with or without gossypol. Genes which varied significantly in Wnt signaling were listed. N number =5.

Mice were injected with gossypol and control vehicle and subjected for RNA-seq analysis. Consistent with our previous publication, Wnt signaling was significantly increased, evidenced by the remarkable upregulation of* Wnt*,* CK1*,* Axin, β-catenin, *and* Cyclin D1*. Interestingly, the upstream gene of Wnt signaling,* WIF1*, was notably decreased (Figure 1), which hinted that gossypol regulated Wnt signaling through WIF1.

### 3.2. Gossypol Treatment Inhibited WIF1 in Mouse Model

**Figure 2:**
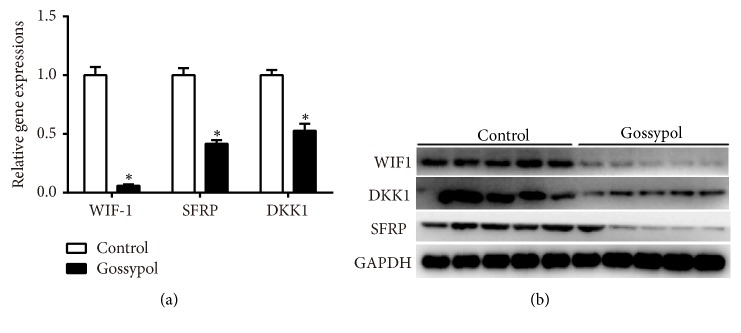
*Gossypol treatment inhibited WIF1 in mouse model*. (a) qRT-PCR assays detected the mRNA levels of WIF1, SFRP, and DKK1 in ovariectomy-induced osteoporosis mice injected with or without gossypol. *∗P<*0.05,* vs.* control. (b) Western blot analysis examined the protein levels of WIF1, SFRP, and DKK1 in ovariectomy-induced osteoporosis mice injected with or without gossypol.

We then extracted mRNA and proteins from each mouse from the mouse model injected with or without gossypol. As shown in Figure 2(a), the mRNA levels of* WIF1, SFRP*, and* DKK1* were decreased remarkably. Of note, the transcript level of WIF1 remained less than 10% after gossypol treatment. Western blot analysis further demonstrated that the protein levels of WIF1, DKK1, and SFRP were notably suppressed by treatment of gossypol (Figure 2(b)). These results together with our former findings showed that gossypol promoted Wnt signaling through inhibiting the expression of WIF1 in ovariectomy-induced osteoporosis.

### 3.3. Gossypol Inhibited the Expression of WIF1 through Its Promoter

**Figure 3:**
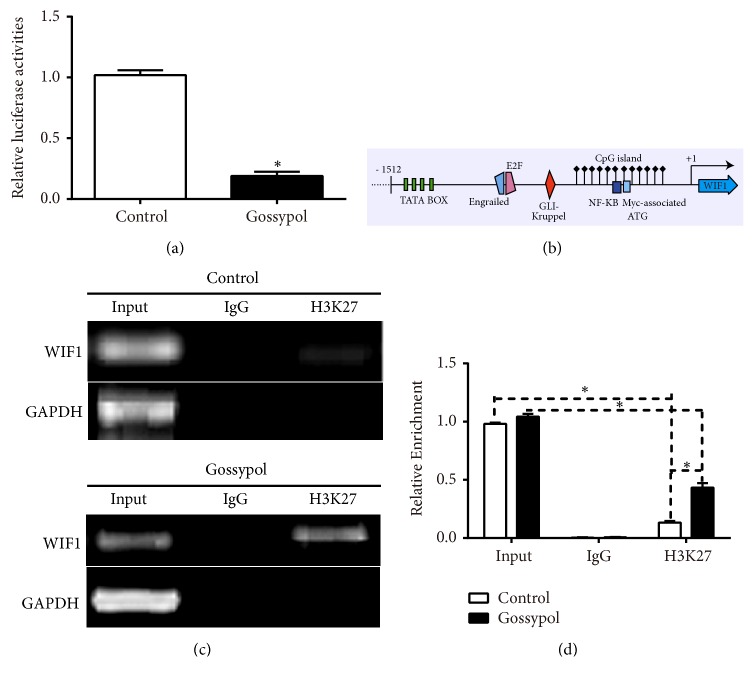
*Gossypol inhibited the expression of WIF1 through its promoter*. (a) Gossypol treatment decreased the luciferase activity of WIF1 in 293T cells. (b) The promoter structure of WIF1 from Atlas database. (c) ChIP-PCR assays detected the effects of gossypol on methylation of WIF1. (d) ChIP-qPCR assays detected the effects of gossypol on methylation of WIF1. *∗P<*0.05,* vs.* control.

Next, we tried to explain the detailed mechanism of the regulatory effect of gossypol on WIF1. To this end, we constructed a luciferase reporter plasmid of WIF1 and transfected it into 293T cells with Renilla control plasmid. As shown in Figure 3(a), the luciferase activity of WIF1 was inhibited by gossypol treatment. By analyzing the structure of WIF1 promoter from Atlas database (http://atlasgeneticsoncology.org/Genes/GC_WIF1.html), the CpG island was found (Figure 3(b)), which was the well-known methylation site. Therefore, we examined the methylation levels of WIF1 and found gossypol treatment increased the methylation level in the promoter of WIF1 significantly (Figures 3(c) and 3(d)). These data showed gossypol suppressed the expression of WIF1 through its methylation site in the promoter of WIF1.

### 3.4. Overexpression of WIF1 Revised the Effects of Gossypol on Cell Proliferation and Cell Apoptosis

**Figure 4:**
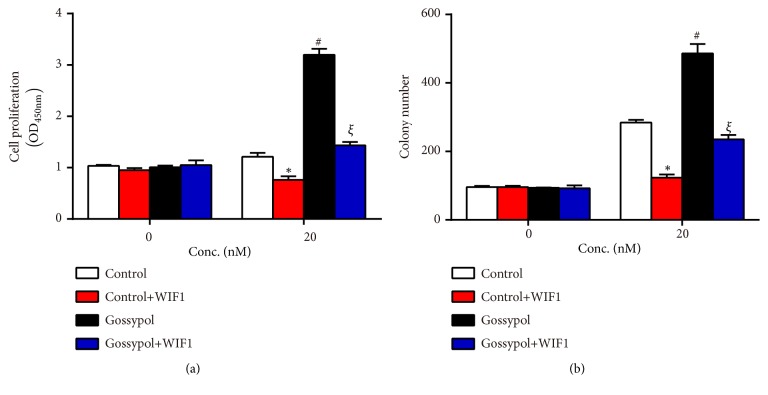
*Gossypol promoted cell proliferation through regulating WIF1 in MC3T3-E1 cells*. (a) Cell viability assays were performed in cells transfected with WIF1 expressing plasmid in the presence or absence of gossypol treatment. (b) Colony formation assays were performed in cells transfected with WIF1 expressing plasmid in the presence or absence of gossypol treatment. *∗P<*0.05, control+WIF1* vs.* control. ^#^*P<*0.05, gossypol* vs.* control. ^*ξ*^*P<*0.05, gossypol+WIF1* vs. *control+WIF1.

**Figure 5:**
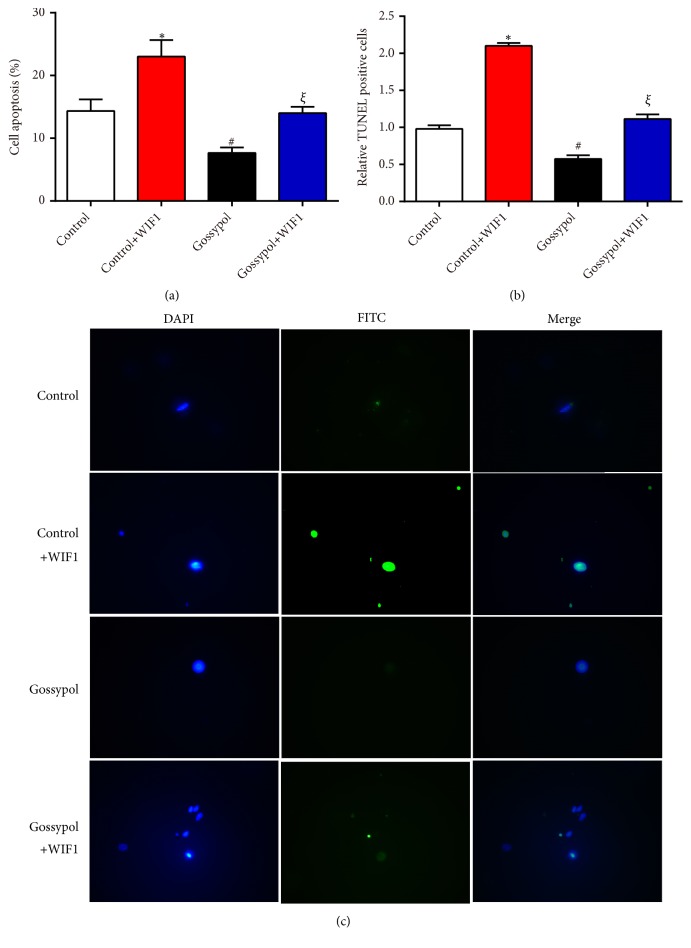
*Gossypol inhibited cell apoptosis via WIF1 in MC3T3-E1 cells*. (a) Cell apoptosis assays were performed in WIF1 overexpression cells with or without gossypol treatment. (b) The quantification of TUNEL assays in MC3T3-E1 cells transfected with WIF1 and treated with gossypol. *∗P<*0.05, control+WIF1* vs.* control. ^#^*P<*0.05, gossypol* vs.* control. ^*ξ*^*P<*0.05, gossypol+WIF1* vs. *control+WIF1.(c) Representative images of TUNEL assays.

MC3T3-E1 cells were transfected with WIF1 expressing plasmid in the presence or absence of gossypol treatment and cell viability in each experimental group was detected. As shown in Figure 4(a), WIF1 expressing plasmid alone suppressed cell proliferation, gossypol treatment increased cell proliferation, and costimulation of gossypol and WIF1 expressing plasmid recovered cell proliferative rate to the normal level. Similarly, colony formation assay also confirmed that WIF1 overexpression retarded colony formation, while gossypol promoted colony formation in MC3T3-E1 cells (Figure 4(b)). Afterwards, cell apoptosis was assessed in MC3T3-E1 cells. As shown in Figure 5(a), WIF1 overexpression increased cell apoptotic rate by 9%, gossypol treatment decreased cell apoptosis by 6%, whereas cotreatment with WIF1 and gossypol made cell apoptosis remain in the normal levels. TUNEL kits were involved to further demonstrate the effects of gossypol on cell apoptosis. Likewise, the TUNEL positive cells increased to two folds of control cells upon WIF1 overexpression and decreased to 56% after gossypol treatment, while no significant difference was observed upon costimulation of WIF1 expressing plasmid and gossypol (Figures 5(b) and 5(c)). These data suggested that gossypol regulated cell proliferation and cell apoptosis through WIF1.

### 3.5. Gossypol Ameliorated Ovariectomy-Induced Osteoporosis through Inhibiting the Expression of WIF1* In Vivo*

**Figure 6:**
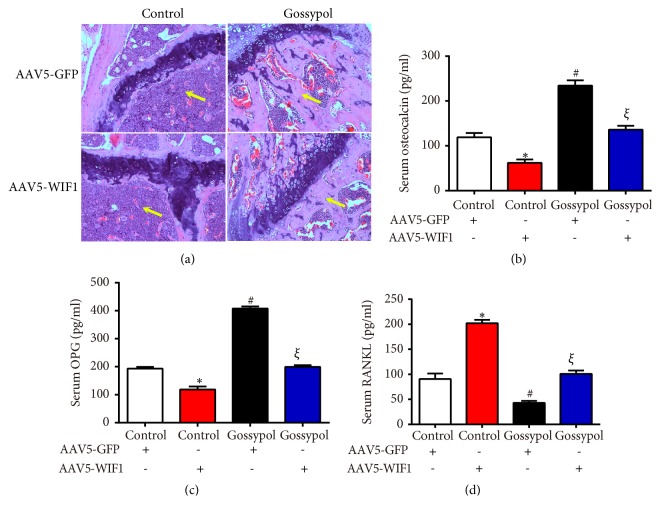
*Gossypol ameliorated ovariectomy-induced osteoporosis through WIF1 in AAV5-injected mice*. (a) Histopathological images of each mouse in the four treating groups. (b) Serum osteocalcin levels in each mouse were assessed. (c) Serum OPG levels in each mouse were detected. (d) Serum RANKL levels in each mouse were examined. *∗P<*0.05, control+WIF1* vs.* control. ^#^*P<*0.05, gossypol* vs.* control. ^*ξ*^*P<*0.05, gossypol+WIF1* vs. *control+WIF1.

To further identify the detailed mechanism of gossypol, ovariectomy-induced osteoporosis mice received an intra-articular injection of AAV5-WIF1 with or without gossypol treatment. Histological analysis showed gossypol injection relieved osteoporosis which was evidenced by a notable increase in metaphyseal regions (yellow arrows) and AAV5-WIF1 injections aggravated osteoporosis. Moreover, the gossypol protective effects were reversed by AAV5-WIF1 injection (Figure 6(a)). Then we detected several serum chemicals with ELISA assays. As shown in Figures 6(b) and 6(c), serum osteocalcin and OPG levels were increased by gossypol and reversed to normal levels when mice were cotreated with gossypol and AAV5-WIF1. However, the serum RANKL levels were decreased by almost 50% of the control mice when gossypol was injected, which was also reversed by further injection of AAV5-WIF1 (Figure 6(d)).

**Figure 7:**
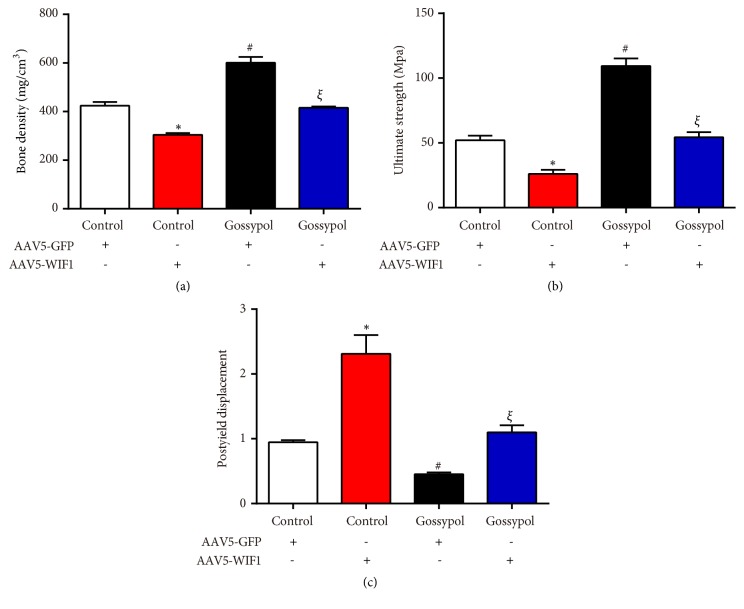
*Gossypol ameliorated ovariectomy-induced osteoporosis evidenced by micro-CT scanning*. (a) Bone density was examined in each mouse. (b) Ultimate strength was detected in ovariectomy-induced osteoporosis mice injected with or without WIF1 and gossypol. (c) Postyield displacement was assessed in ovariectomy-induced osteoporosis mice. *∗P<*0.05, control+WIF1* vs.* control. ^#^*P<*0.05, gossypol* vs.* control. ^*ξ*^*P<*0.05, gossypol+WIF1* vs. *control+WIF1.

**Figure 8:**
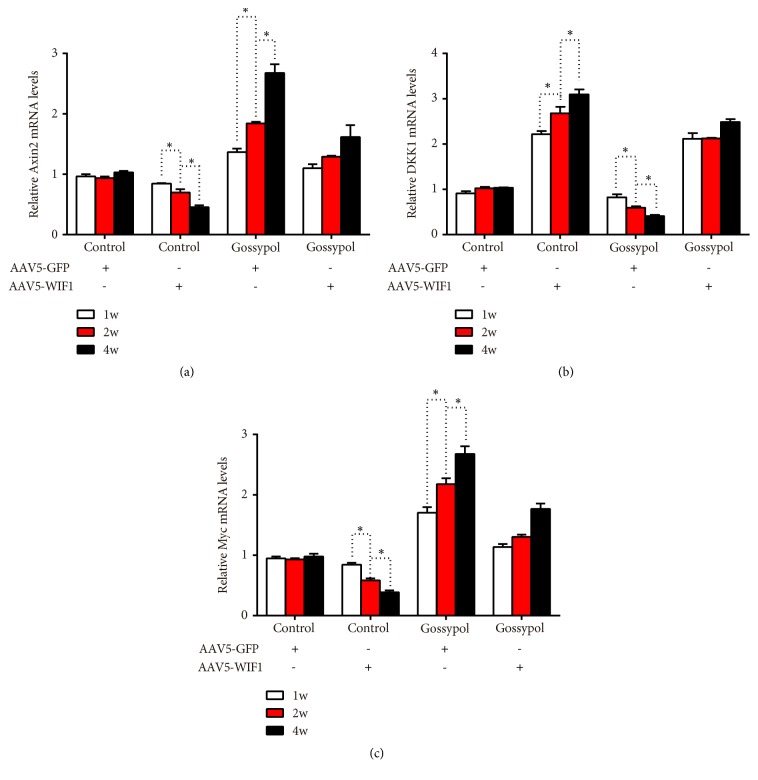
*Gossypol ameliorated ovariectomy-induced osteoporosis evidenced by qRT-PCR analysis*. (a) mRNAL levels of Axin2 in mice injected with or without WIF1 and gossypol. (b) mRNAL levels of DKK1 in mice injected with or without WIF1 and gossypol. (c) mRNAL levels of Myc in mice injected with or without WIF1 and gossypol. *∗P<*0.05, as indicated.

Next, micro-CT scanning was conducted in each mouse and the results were consistent with the former findings. Gossypol-injected mice had higher bone density than the control mice; however, after being coinjected with AAV5-WIF1, the bone density recovered to the control levels (Figure 7(a)). The ultimate strength in control mice was 50Mpa, gossypol injection increased ultimate strength to approximately 100Mpa while AAV5-WIF1 injection decreased ultimate strength to almost 25Mpa and coinjection with gossypol, and AAV5-WIF1 abolished the protective effects of gossypol on ultimate strength (Figure 7(b)). Moreover, the postyield displacement of gossypol-injected mice was dropped to 50% and increased to 200% upon AAV5-WIF1 injection and further recovered to control levels upon gossypol treatment and WIF1 overexpression (Figure 7(c)). Furthermore, related genes were detected with qRT-PCR analysis. As shown in Figure 8, AAV5-WIF1 inhibited the mRNA level of Axin2 and Myc while promoting that of DKK1 in a time-dependent manner; however, these effects were revised by cotreatment with gossypol. These results together demonstrated gossypol promoted bone formation via inhibiting the expression of WIF1 in ovariectomy-induced osteoporosis.

## 4. Discussion

Numerous studies demonstrated that ovariectomy-induced hormone deficiency negatively affects study and memory as well as bone formation [10].

Three Wnt signaling pathways have been identified, which are the canonical Wnt pathway, the noncanonical cell polarity pathway, and the noncanonical Wnt/calcium pathway, all of which are activated by the binding of Wnt ligand to a Frizzled family receptor. Wnt signaling pathways are highly evolutionarily conserved and play significant roles in carcinogenesis [11–13], embryonic development [14], diabetes [15–17], and osteoporosis [18]. To ensure proper functioning, Wnt signaling is regulated constantly. For instance, the secretion of Wnt proteins was controlled by GPR177 and retromer complex [19]. After secretion, the ligand can be prevented from binding to its receptor, such as Dally and Glypican 3. DKK, WIF1 [20], SFRP, SOST, and Naked cuticle are also specific antagonists [21, 22]. Our study found gossypol regulated Wnt signaling through methylation of WIF1. When WIF1 was overexpressed* in vivo* and* in vitro*, the promotion of Wnt signaling by gossypol was revised, evidenced by normal cell proliferative rate and cell apoptotic level.

With the use of RNA-seq, the Wnt inhibitory factor 1 (WIF1) was identified to be a critical factor responding to gossypol treatment. WIF1 is a Wnt signaling antagonist that directly interacts with various Wnt ligands and inhibits their binding to membrane bound receptors [23]. In particular, hypermethylation of the WIF1 promoter, leading to WIF1 silencing (and thus activation of Wnt/beta-catenin signaling), was shown to be associated with various types of cancers including osteosarcoma [24], which indicated the association of WIF1 with osteoporotic diseases. In the present study, we have found that gossypol treatment significantly suppressed the expression of WIF1 at both the mRNA and the protein levels, leading to the enhancement of Wnt/*β*-catenin signaling. Meanwhile, gossypol regulated the methylation levels of WIF1 to regulate its expression, which was consistent with previous reports [23]. As a consequence, the gossypol-mediated protection against osteoporosis was significantly blunted after overexpression of WIF1 through a genetic approach. It is therefore conclusive that gossypol protected against osteoporosis via inhibiting the expression of WIF1.

The identification of the Wnt signaling antagonist WIF1 as a target of gossypol is of great biological importance. On one hand, this finding adds knowledge to the molecular mechanisms underlying gossypol biological function. Previously we reported the protective effects of gossypol on osteoporosis by enhancing Wnt signaling [9]. The current study further elucidated the inner link between gossypol and the Wnt signaling. On the other hand, the WIF1-dependent protection effects of gossypol suggested that patients with WIF1 suppression should yield satisfactory treatment efficacy. We added the clinical values that gossypol might be beneficial in patients with the evaluated low expression level of WIF1 which are supposed to show high response to gossypol treatment.

## 5. Conclusion

In all, the present study identified WIF1 as a critical target of gossypol by which gossypol promoted cell apoptosis and inhibited cell proliferation. Gossypol might be effective in treating osteoporotic patients with evaluated low expression of WIF1 in clinic. Our results provided deeper data of the protective roles of gossypol and might pave novel ways for the clinical treatment of osteoporosis.

## Data Availability

The data used to support the findings of this study are available upon request.
